# Guilty repair sustains cooperation, angry retaliation destroys it

**DOI:** 10.1038/srep46709

**Published:** 2017-04-27

**Authors:** Anya Skatova, Alexa Spence, Caroline Leygue, Eamonn Ferguson

**Affiliations:** 1 Warwick Business School, University of Warwick, Coventry, UK; 2 Horizon Digital Economy Research, University Of Nottingham, Nottingham, UK; 3 School of Psychology, University Of Nottingham, Nottingham, UK

## Abstract

Sustained cooperative social interactions are key to successful outcomes in many real-world contexts (e.g., climate change and energy conservation). We explore the self-regulatory roles of anger and guilt, as well as prosocial or selfish social preferences in a repeated social dilemma game framed around shared electricity use at home. We explore the proposal that for sustained cooperation, guilty repair needs to override angry retaliation. We show that anger is damaging to cooperation as it leads to retaliation and an increase of defection, while, through guilt, cooperation is repaired resulting in higher levels of cooperation. We demonstrate a disconnect between the experience of anger and subsequent retaliation which is a function of participants’ social preferences. While there is no difference in reports of anger between prosocial and selfish individuals after finding out that others use more energy from the communal resource, prosocials are less likely to act on their anger and retaliate. Selfish individuals are motivated by anger to retaliate but not motivated by guilt to repair and contribute disproportionately to the breakdown of cooperation over repeated interactions. We suggest that guilt is a key emotion to appeal to when encouraging cooperation.

Understanding what motivates people to cooperate and act in the interest of the group is vital for human society. It has relevance to policy-makers facing the challenges of climate change, incurable diseases, military conflicts and failing economies. While traditional economic models of utility maximization cannot fully explain cooperation^1^,^2^,^3^,^4^, recent research suggests that emotions associated with concern for others and for the group can help to sustain cooperation^2^,^5^,^6^. According to strong reciprocity theory, anger encourages punishment of transgressors in order to maintain cooperation^7^,^8^,^9^. However, scenarios where punishment of transgressors is possible describes only a subset of cooperative situations. In many real world cooperative situations direct punishment of transgressors is not possible^10^ and/or other options to react to behaviour of others are available, such as to reward peers’ cooperation^11^,^12^,^13^. One way of encouraging cooperation is to regulate one’s own behaviour to maintain cooperation, and emotions are seen as offering a mechanism to achieve this^14^. Unlike previous research, which examined the role of emotions in scenarios with punishment or venting opportunities^15^,^16^,^17^, the current study examines a more common function of emotions, as a self-regulating endogenous mechanism to motivate changes in cooperative behaviour. This is modelled around a shared resource when peer-punishment is not possible, specifically focusing on the role of emotions in reparation and retaliation^1^,^18^.

Research on why people cooperate often uses social dilemmas such as public goods games as an experimental model of cooperation^12^,^19^,^20^. In a public goods game players are presented with a conflict between the opportunity to gain benefits through cooperation in a group or to receive individual profits, which often comes at some cost to others. If everybody cooperates, all group members are better off, but individual members do not know the degree to which others will cooperate^21^. This creates uncertainty about how to behave. Responding to emotions generated by others’ actions or one’s own actions is one way to deal with this uncertainty. Subsequent reflection on personal actions, or actions of others, prompts emotional responses which lead individuals to adjust their behaviour. Anger, on the one hand, can enforce cooperation through mechanisms of altruistic punishment (when punishment opportunities are present)^7^,^22^. On the other hand, anger is also linked to retaliation^2^ and revenge^23^, creating an escalation of conflicts. Escalation of non-cooperation, due to anger, may be especially likely when sanctioning possibilities are limited or do not exist^23^,^24^,^25^. The negative consequences of endogenous anger on group outcomes during repeated interactions in cooperative games have not been examined. Furthermore, to understand cooperation in small groups, it is important to consider not only how people react to other’s unfair behaviour, but also how people react to their own unfair behaviour. Often when people realise they have acted unfairly, they feel guilt, which leads to reparation^26^. However, the longer term effects of guilt in cooperation – over a number of repeated interactions - have not been studied. One clear gap in the literature is whether reparation, motivated by guilt at one’s own unfairness, is sufficient to sustain cooperation. Indeed, guilt may lead to the repair of cooperation without the potential negative effects of anger^27^. Given that there are always differences in the amounts that individuals contribute to a public good or take from a shared resource, it is essential that those who cooperate less subsequently repair, if cooperation is to be sustained. Thus to sustain long-term cooperation, guilty repair needs to override angry retaliation^26^,^28^,^29^. In this experiment we studied the effects of individual-level anger and guilt beyond one-shot interactions, their role in repair and retaliation, and their effect on group level cooperation over repeated interactions. Answering these questions can help understand the role of emotions in cooperation and how the dynamics of cooperation and unfairness in groups depends on individual emotional responses.

We also explore the role of individual differences in emotional responses and decision making^18^. Not everybody will experience guilt and anger to the same extent -when facing the same event some individuals will feel a lot of guilt or anger and some none at all- and/or not all will act on their experienced emotions. In addition, research shows that the way individuals choose to play at the onset - to be fair and contribute, or to free ride - influences their response to others’ actions^30^. Thus in this study, we considered how individual behaviour and emotional responding varied depending on social preferences, demonstrated by initial indication of prosocial or selfish preferences. This is important as we know that those who have a tendency towards prosocial preferences cooperate more, and by implication should conserve more resources. One mechanism for sustained cooperation is through individual response to the feelings of guilt and anger. We propose that those who do not feel guilty or feel low levels of guilt are less likely to repair and in such groups cooperation will decline and free riding will escalate. Furthermore, in groups where individuals feel higher levels of guilt, repair is more likely, and for those groups where reparation operates, cooperation should be sustained. We predict that individuals who indicate prosocial preferences and cooperate initially, compared to those who have selfish preferences, are likely to feel greater guilt and also more likely to act on that guilt and repair to maintain cooperation. In addition, those individuals would be less likely to feel anger, and also less likely to act on their anger by retaliating. We refer to the emotion regulation mechanism for sustaining cooperation as ‘guilty repair overrides angry retaliation’.

Economic game methodology and associated vignette studies^31^ allow for simulating cooperative processes within real world scenarios. Such methodologies often generalise well to real world behaviour^31^, and real world situations are hard to explore in depth due to the complexity of parameters and reporting biases. Thus we used a repeated negatively-framed public goods game^32^ to investigate the role of emotions in cooperation. Previous research has demonstrated the utility of modelling environmental choices using social dilemmas^33^,^34^,^35^,^36^, however laboratory public goods games have not previously been used to examine cooperation around a shared energy resource. Our design is an advance over standard psychological designs, as instead of focusing on perceptions or behavioural intentions we investigated actual behaviour during real time social interactions. We specifically link monetary units and participation rewards to energy use and set the scenario within a house share situation. Thus, our design provides a higher degree of realism with respect to social interactions than traditionally in psychology and is closer to real world decisions than traditional economic games.

We chose a shared household scenario as it provides a realistic framing for our participants – mainly students. A house share is a context that is common for many people where there is a ground rent; it is also a scenario that may be extended to shared office spaces where energy bills are charged based on floor-space rather than energy use. The public goods game we used was presented to participants in a context of a house-sharing situation (see Fig. 1 for details and the scheme of one round of the game), where individuals interacted with each other over a restricted household electricity resource, and where individual electricity usage was fed back to participants at the end of each period. In this scenario, the feedback about behaviour of others is the feedback about communal energy use. This is relevant to the rollout of smart meters – ongoing in the UK and many other countries around the world^37^ - and the accompanying increasing use of energy displays, both domestic and commercial. Advances in disaggregation of electricity use means that users are able to understand better the electricity they consume individually and relative to other people^38^, meaning that energy sharing situations at home resemble a social dilemma.

118 participants took part in randomly allocated groups of four in two blocks of ten rounds where they remained anonymous to one another but kept the same partners within each block. Of these 118 data was analysed from 72 and 76 in Blocks 1 and 2 respectively – see Methods for details. Partners in the groups were switched for the second block. Participants were asked to imagine they were sharing a house with three others who they did not know (cf. a bedsit, university flat). They were given an initial endowment of 20 Monetary Units (MU) and required to make a decision on how much electricity they were going to use (usage limits were from 5 to 20 Electricity Units, EU). Payments for the resource were shared at the end of each round. Every 2 EUs used earned the individual player 1 MU of individual profit reflecting the utility of light, heat and other benefits that people would normally receive from electricity use. After participants’ private decisions about electricity use for the current round were made, they shared the bill equally regardless of individual use. The game design was such that if everyone used an equal amount of electricity the outcome would be fair. However, if a participant used more than their partners, they would receive more utility (translated into real monetary rewards) and if they underused, they would receive less. This situation is reflective of real life: for example in shared accommodation bills are often divided equally even though usage is likely to differ, and those who use more can gain more utility. Following each round, participants rated to what extent they felt the key emotions of anger and guilt, amongst other emotions (included to avoid demand effects), on a scale from 1 (not at all) to 7 (extremely).

## Results

### Cooperation and Retaliation

**Figure 2:**
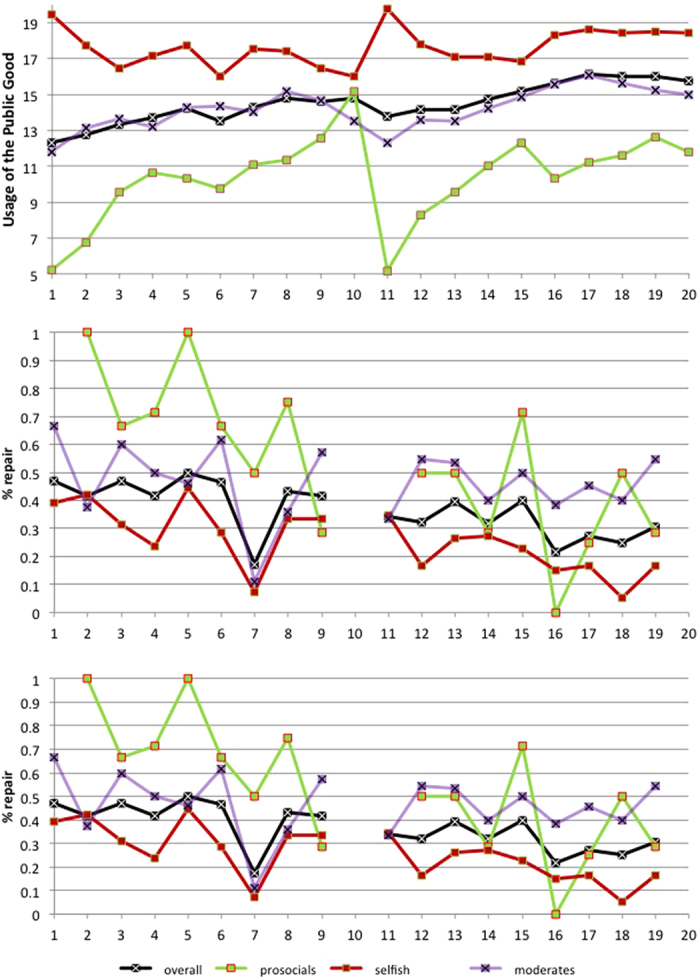
Usage of the Public Good, levels of conditional retaliation and repair. (**a**) Mean usage. (**b**) Percent retaliation when others used more. (**c**) Percent repair when others used less. Lines illustrate overall usage (black), moderates (purple), prosocials (green) and selfish (red) over 10 rounds of Block 1 (Rounds 1–10) and 10 rounds of Block 2 (Rounds 11–20). Retaliation was defined as increased usage on round *n* + *1* after finding out that others used more than the participant themselves on round *n*. Repair was defined as decreased usage on round *n* + 1 after discovering that others used less than the participants themselves on round *n*.

**Figure 3:**
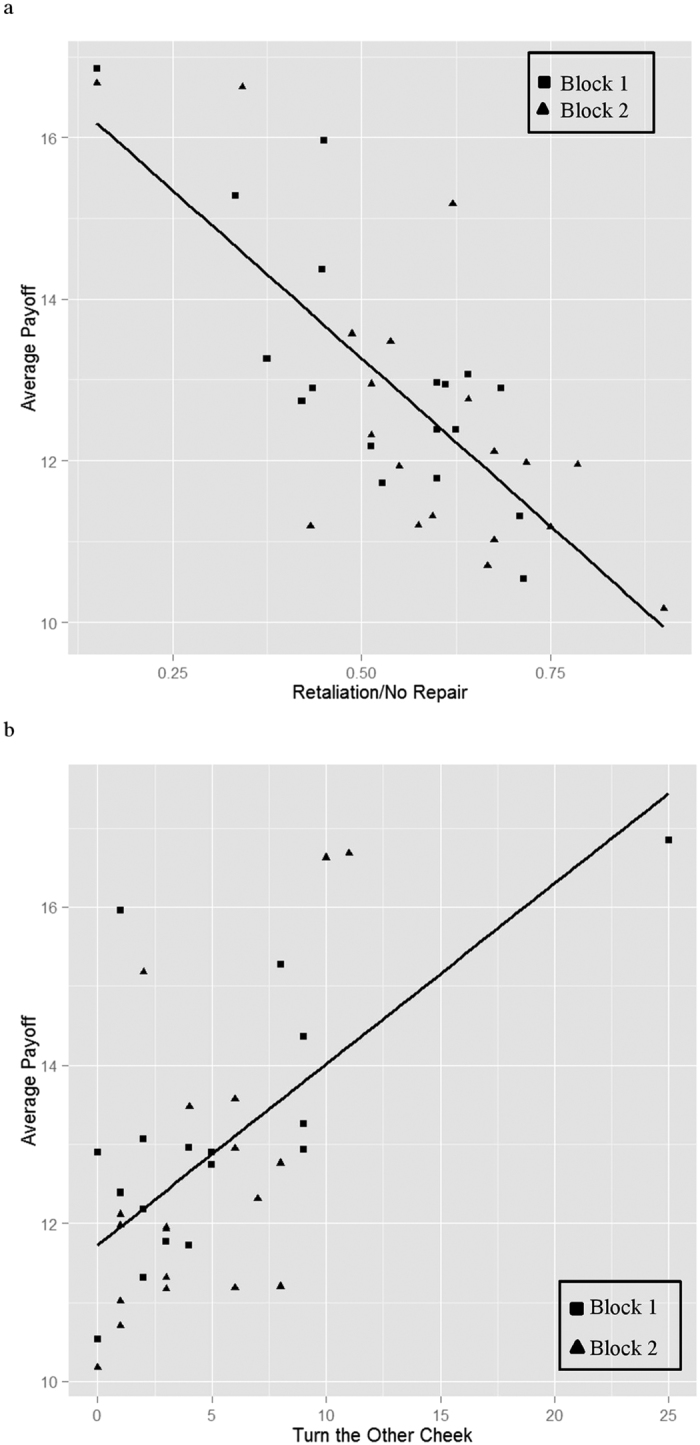
Average group-level payoffs in both blocks. (**a**) Payoff against frequency of conditional (after finding out that others used more) retaliation and non-repair in the group. (**b**) Payoff against frequency of non-retaliation or ‘turning the other cheek’ towards transgressions of others across two consecutive rounds (in round *n* and *n* + *1*). Groups with a high level of conditional retaliation and no repair made less profits overall, while groups that used a “turn the other cheek” strategy gained more.

Defection increased over the rounds in each block (Fig. 2a, black line): usage in Block 1, Round 1 was significantly lower (M = 12.64 EUs, SD = 5.83 EUs) than usage in Round 10 (M = 14.79 EUs, SD = 5.39 EUs): t_71_ = −2.78, *p* = 0.007, paired-sample; usage in Block 2, Round 1 was significantly lower (M = 13.64 EUs, SD = 5.81 EUs) than in Round 10 (M = 15.64 EUs, SD = 5.63 EUs): t_75_ = −3.001, *p* = 0.004, paired-sample. All p-values reported here and below throughout the paper are for two-tailed tests. The increase of defection in this case implied a rise in retaliation and lack of repair as depicted in Fig. 2b,c, black lines. Retaliation (repair) was defined as an increase (decrease) in use in response to higher (lower) use of others (see Method for calculations of indices). Furthermore, retaliation was detrimental to overall payoffs: group earnings were negatively affected by retaliation and non-repair in both blocks (OLS, slope = −8.56, *p* < 0.001 for Block 1, slope = −8.09, *p* < 0.001 for Block 2, Fig. 3a). The levels of retaliation were higher than the levels of repair overall: in 57% of times when others free rode participants retaliated, whilst they repaired in response to behaviour of others in only 36% of cases when they themselves used more than others; this corresponded to an increase in non-cooperation over the block. In addition, groups that retaliated more and repaired less overall, yielded lower profits (Fig. 3). Further, the groups where participants demonstrated “turn the other cheek” behaviour (i.e., did not retaliate after two rounds of uncooperative behaviour of their group), saved more electricity than groups with less patient participants (OLS, slope = 0.18, *p* = 0.004 for Block 1, slope = 0.36, *p* = 0.002 for Block 2; Fig. 3b). These data were analysed at the level of the group to account for interdependence of outcomes for members of each group^12^.

In addition, our data demonstrated a “restart effect” consistent with the literature^2^. There was no significant difference between usage in Round 1, Block 1 and Round 1, Block 2, t_145_ = 1.38, *p* = 0.17, paired-sample: when moving to play with a new group in Block 2 participants dropped their electricity usage to the same level as in Round 1, Block 1. This shows that participants did not simply increase their electricity usage to improve their profits in the study, but rather changed their behaviour in reaction to the behaviour of others in their group.

### Emotions and Cooperation

Self-reported anger did not change significantly over the rounds in Block 1 but in Block 2 it decreased between the first and last rounds: Round 1 (Block 1: M = 2.76, SD = 1.66; Block 2: M = 2.93, SD = 1.91) and Round 10 (Block 1: M = 2.81, SD = 1.85; Block 2: M = 2.42, SD = 1.74): paired-sample, Block 1: t_71_ = −0.35 ns; Block 2: t_75_ = 2.19, *p* = 0.03. Guilt marginally increased in the last round of Block 1, compared to the Round 1, but did not change significantly over the rounds in Block 2: Round 1 (Block 1: M = 1.60, SD = 1.00; Block 2: M = 1.79, SD = 1.37) and Round 10 (Block 1: M = 1.88, SD = 1.39; Block 2: M = 2.08, SD = 1.80); paired-sample, Block 1: t_71_ = −1.84, *p* = 0.07; Block 2: t_75_ = −1.29 ns. We tested if there was any change in guilt and anger over time, conditional on the fair or unfair behaviour of others in the group using mixed-effects regressionthat modeled participants’ random intercepts and slopes for unfairness of others and round number (see Method for details of analysis strategy and Supplementary Table S1 for full results), with guilt and anger as outcomes. These showed that fairness of others (when others use less than the participant, defined as a negative deviation from the mean of three other players) predicted increased guilt: B = −0.09, 95% Confidence Intervals (CI): [−0.13; −0.06], *p* < 0.001; while unfairness of others (when others use more than the participant, defined as a positive deviation from the mean of the other players) predicted increased anger B = 0.14, 95% CI: [0.12; 0.17], *p* < 0.001. Further, mediation analyses (see Supplementary Fig. S1 for the model), with energy use on the next round as an outcome, showed that guilt mediated effects of fair behaviour of others and caused a decrease in energy use with a direct effect of guilt B = −0.24, 95% CI: [−0.43; −0.04], *p* = 0.018, and with the proportion of variance mediated by guilt being 0.04, 95% CI: [0.01; 0.09], *p* < 0.001. Anger showed a marginal direct effect on increase in energy use (B = 0.15, 95% CI: [−0.10; 0.24], *p* = 0.08), and also significantly interacted with the unfairness index: B = 0.05, 95% CI: [0.03; 0.08], *p* < 0.001 (see Supplementary Materials for details of the analyses), so that anger amplified participants’ increase in energy use in response to unfair behaviour of others.

### Social Preferences

To investigate these dynamics further, we looked for differences in interaction strategies. We extracted three social preference strategies that could have had an effect on the dynamic social interactions, namely prosocial, moderate and selfish preferences. Individuals identified as prosocials used at least one standard deviation below the mean usage on the first round of the block or 7 EUs or below. Those who were identified as having selfish (profit-seeking) preferences initially played at least one standard deviation above the mean usage on the first round, or 18+ EUs. All participants between these values were classified as moderates. This classification also converged with an independent measure of social value orientation (see Method). Prosocials, moderates and selfish individuals showed different patterns of behaviour in response to the behaviour of others: prosocials used less than others and were, in general, fair compared to others, with mean difference between their own use and the use of the group being 1.71 EUs (SD = 5.33). Moderates in general used slightly less than the group (M = 0.33, SD = 4.10), while selfish individuals, on average, exploited the group: M = −1.57, SD = 5.47. The difference between three groups were significant: *F*_*[2,1477]*_ = 2462.78, *p* < 0.001. Pairwise comparisons showed significant differences between prosocials and the moderates, prosocials and the selfish, and moderates and the selfish at p < 0.001 level. In terms of the effect of different social preferences on group-level behaviour, we observed that in groups with three or more prosocials profits were significantly higher (16.29 MUs on average) than in groups with three selfish players (11.22 MUs on average): *t*_*3.71*_ = −10.08, *p* < 0.001.

### Preferences, Emotions and Cooperation

Mixed-level random effects models demonstrated that prosocials, moderates and selfish players differed with respect to their emotional profiles in expected directions: as predicted prosocials reported more guilt in general (B = 0.25, 95% CI: [0.02; 0.49], *p* = 0.03), while moderates reported more instrumental guilt, or guilt after finding out that they used more than others (B = −0.09, 95% CI: [−0.16; −0.03], *p* < 0.001). Both prosocials and moderates reported less anger in general (B = −0.57, 95% CI: [−0.83; −0.32], *p* < 0.001 and B = −0.44, 95% CI: [−0.67; −0.22], *p* < 0.001, respectively), while moderates reported marginally more instrumental anger, or anger after finding that others used more: B = 0.06; 95% CI: [−0.001; 0.11], *p* = 0.06. Everybody reported feeling angry when facing unfair behaviour of others: the effect of unfairness on anger remained significant controlling for social preferences: direct effect of unfairness on anger was positive and significant B = 0.11, 95% CI: [0.08; 0.16], *p* < 0.001 (see Supplementary Fig. S3 for the model specification).

**Figure 4:**
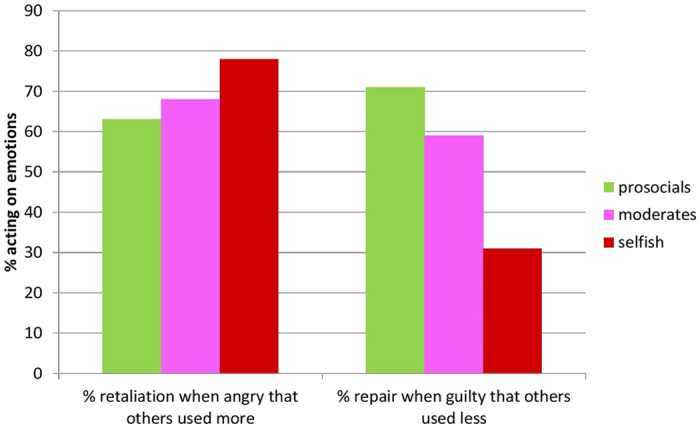
Angry retaliation and guilty repair. Percentage of cases when acting upon one’s emotions.

Furthermore, as predicted, guilt motivated behaviour to repair in prosocials and moderates only (see Supplementary Fig. S2 for the model specification): B = −0.67, 95% CI: [−1.23; −0.10], *p* = 0.02; B = −0.87, 95% CI: [−1.33; −0.41], *p* < 0.001, respectively. In addition, moderates and prosocials acted less on their anger than selfish players when others were unfair. When feeling angry, prosocials and moderates increased their energy use but by less than selfish players (B = −0.09, 95% CI: [−0.17; −0.02], *p* = 0.04 and B = −0.17, 95% CI: [−0.24; −0.10], *p* < 0.001, respectively). Prosocials acted on anger marginally less than moderates (pairwise comparisons of beta coefficients were at *p* = 0.08). We also looked in more detail at retaliatory and reparatory behaviour as trigged by anger and guilt across the three social preference groups. There were no significant differences for angry retaliation across preferences; on average, prosocials showed angry retaliation in 63% of cases, moderates in 68% and selfish players in 78% of cases, F_*[2,75]*_ = 1.57, ns, see Fig 4. The differences in guilty repair were, however, significant with prosocials repairing in 71% of cases, moderates in 59% and selfish players in only 31% of cases, F_*[2,64]*_ = 6.54, *p* < 0.001. Pairwise comparisons revealed significant differences in guilty repair between selfish players and prosocials (*p* < 0.001) and selfish players and moderates (*p* = 0.03).

## Discussion

We demonstrate that in a repeated-interaction scenario, where people cooperate around a shared resource, cooperation breaks down and the use of the public good spirals upwards leading to poorer outcomes for the group. This is consistent with previous research on cooperation^3^,^39^. Our paper makes three main contributions to the literature. First, we show that retaliation and non-repair is a process through which cooperation around a shared resource in a repeated interaction scenario breaks down. Second, we demonstrate that guilt mediates cooperation through repair, while anger moderates the breakdown of cooperation through retaliation. Third, we show that a group of selfish individuals who are motivated by anger to retaliate and are not motivated by guilt to repair is a key reason why cooperation is not sustainable within repeated interaction scenarios. We highlight a disconnect between the experience of anger towards free-riding and subsequent behaviour as a function of social preferences (prosocial, moderate or selfish). We show that while everybody reports anger after being exploited, prosocials and moderates, compared to players with a selfish preference, are less likely to act on their anger and retaliate, but are more likely to show guilty repair if they overused from the resource themselves (guilty repair overrides angry retaliation). It is the existence of selfish individuals, whose angry retaliation is greater than any guilty repair they demonstrate, that is why interactions result in the breakdown of cooperation.

We find that higher levels of reparation (i.e., decrease in energy use in response to cooperative behaviour of others), and non-retaliation led to greater group profits, in our case energy savings, whilst acting upon anger was detrimental to cooperation. Anger towards free-riding led to retaliation (i.e., increase in energy use in response to uncooperative behaviour of others). This is in line with previous findings^40^ but had not been previously applied to repeated same partner interactions. We acknowledge that anger could have also been caused by perceived unfairness to others (e.g., moral outrage if I see that one of the group partners is free-riding while the other one is over-contributing), and future research could investigate whether the group average or, alternatively, different distributions of use by others affect different individual emotions. We are the first to demonstrate that guilt, as a mechanism to repair cooperation in a public goods game, is especially observed in those with prosocial preferences. We also contribute to the literature on heterogeneity in social dilemmas and prosocial behaviour in general by showing that prosocial preferences can be associated with specific emotional-behavioural profiles in terms of anger and guilt. A decline in cooperation was exacerbated by selfish players who focused on individual level profits, escalated energy use which was motivated by anger, and did not repair to the same extent. Conversely, prosocials and moderates, were more likely to use cooperative strategies and to show concern for energy saving and others’ outcomes (acting on guilt after they benefited at others’ expense)^18^. Importantly, our data contradicts the idea that prosocial individuals do not feel angry with free-riders^18^. We instead find no differences in reports of anger when facing transgressions of others by prosocials, moderates and selfish players, however there are differences in behaviour, with prosocials and moderates acting less on their anger. Thus, there is a disconnect between the experience of emotions and behaviour dependent on social preferences: an increase in anger will not necessarily lead to behaviour change for all in the same way. Importantly, we showed that the strategy of less retaliation and more repair was more useful in sustaining cooperation in the long run^41^.

This data also highlights the need for the consideration of dynamic interactions around the use of energy resources as these are often shared at a household, community, or at a broader level. Given standard premises of game theory, our findings should be generalizable to interactions between households, companies or even countries, and whilst this is yet to be tested, it is a promising avenue to explore in relation to environmental issues such as climate change mitigation^34^. For example, can guilt inducing messages be used as a deterrent of defection on the country-level, and promote cooperation around climate change? Process models of cooperation are needed in order to better forecast social responses to new energy policies, technologies and systems, and to help explain and predict potential heterogeneity in responses observed^42^,^43^,^44^. For example, our results imply that increased visibility of energy use through smart meters may have previously unanticipated impacts due to social interactions around energy use; given that some people will always use more than others, there is a risk of a breakdown of cooperation and energy use spiralling upwards.

We showed that guilty repair, in particular, is a key mechanism for sustaining cooperation, but likely to be observed more in those with prosocial preferences. Thus targeting moral emotions such as guilt in communications and interventions aiming to promote cooperation – such as around communal energy use and in other scenarios where cooperation is key – is likely to be fruitful, especially when the social structure does not provide easy opportunities to sanction free-riding. Indeed, there is evidence that inciting mild anticipatory guilt via framed messages leads to reported increase of targeted prosocial acts such as blood donation^45^. Our research demonstrates that during repeated interactions around a shared resource, where sanctions are not available, the best strategy both personally and for the group, is to cooperate, to avoid retaliation, and repair when necessary.

## Methods

### Participants and procedures

118 participants took part in the study, out of which 113 reported their age and gender: the sample had a mean age of 21.43 years, ranging from 18 to 51; 61.93% were female. Participants were recruited through a university student pool. Participants took part in the study sessions in groups of between 6 to 20 people at a time. They received a monetary reward at the end of the experiment based on the sum of their individual earnings. Earnings were on average £5.80 (equivalent to ~9.33 USD at the time of the study), and ranged from £4.80 to £6.90. Participants had five practice rounds before the start of the experiment to ensure they fully understood the rules of the game. In addition, participants filled in a Social Value Orientation questionnaire (SVO)^46^ that identified individuals with prosocial tendencies. This scale is predictive of behaviour both in the laboratory and the real world. The questionnaire contains nine choices in which participants hypothetically choose from a distribution of payoffs which are either selfish (e.g., benefit oneself) or prosocial (e.g., equally benefit oneself and the other). The number of prosocial choices in each category allowed the classification of participants as being prosocially oriented or not.

We additionally manipulated whether participants received feedback detailing the average electricity use of the rest of their partners (private condition) or received feedback on the electricity use of each individual group partner (public condition). In the “public” condition individual usage was identified by a participant number (therefore still maintaining anonymity). 47% of participants took part in the private condition and the rest in the public condition. As there were no overall differences in electricity use, anger or guilt between private (electricity: M = 14.4 EUs, SD = 5.33 EUs; anger: M = 2.6, SD = 1.73; guilt: M = 1.65, SD = 1.26) and public (electricity: M = 14.83 EUs, SD = 5.21 EUs; anger: M = 2.68, SD = 1.75; guilt: M = 2.11, SD = 1.58) conditions (mixed-effects regression controlling for random participant-level effects; electricity: slope = 0.26, *p* = 0.75; anger: slope = 0.04, *p* = 0.89; guilt: slope = 0.36, *p* = 0.08), for all further analyses these data were combined across conditions. Mean (M) electricity use across conditions was 14.63 EUs, with a SD of 5.32.

Participants entered all responses individually via computers using Z-Tree software^47^ and thus did not know who else in the room was in their group. When full groups of four could not be formed as not all participants who signed up for the experiment showed up, the experimenter joined the game for the rest of participants, each time entering an electricity use of 10 EUs. Since the researcher had to use their computer to operate the program in all sessions, and all participants and the researcher were seated in separate computer booths, this strategy was undetectable to participants. In 63% of cases, individuals made responses without imposters in the group, in 31% there was one imposter in the group, in 4% - 2, in 1% - 3. Out of 118 participants, 72 in Block 1 and 76 in Block 2 played in groups without imposters. Only groups without imposter data were included in analyses. The experiment took approximately one hour for each participant. At the end of the experiment, participants were paid individually. The study was approved by a School of Psychology, University of Nottingham Ethics Committee and was conducted in accordance with relevant guidelines and regulations. Informed consent was obtained from all participants.

### Analysis strategy

#### Mixed-level regression modelling, overall sample

The information about behaviour of others constituted the *unfairness* predictor, which was modelled both on population and participant level: a difference between participants’ own use and the average use of their three other group members on a given round. To study whether information about behaviour of others – fair or unfair - predicted certain emotional reactions, as well as if emotions predicted behaviour on the next round, we conducted a mixed-level random intercept (by participant) and slope (by unfairness and round number) regression using lme4^48^ and mediate^49^ packages in R. First we tested whether emotions mediate or moderate the effect of unfairness on future energy use. Due to the structure of the game, participants first made the decision about their use and then they found out how much others used. Thus, they would see that others were either less fair than them on average, the group used exactly the same or the group was unfair and used more than them on average. The unfairness predictor was calculated so that positive values indicated unfair behaviour of the group, while zero or negative values indicated fairness. We first tested whether anger and guilt was predicted from unfairness, controlling for round number, both on a population and an individual level, to capture repeated-interaction effects (see Supplementary Table S1, Model 1 g and Model 1a). Next, we explored the mediation and moderation effects of emotions on behaviour (see Supplementary Table S1, Models 2, 3 g, 3a, 4 g and 4a). We estimated the unmediated and mediated effect of unfairness on the increase in energy use in the next round controlling for round number, and interactions between unfairness and emotions. Full results of regressions are reported in Supplementary Tables S1 and discussed in Supplementary Materials.

#### Prosocial preferences

To examine the impact of different behavioural strategies in the group we extracted prosocial, moderate and selfish preferences in each block based on the usage in the first round. The use of a small amount at the start of an interaction (in our case below one standard deviation or 7 EUs or less) conveys prosocial preferences. Likewise, a high usage at the start indicates selfish preferences and we labelled all the rest (those, who used between 7 and 18 EUs) as moderates. To ensure that our classification was not biased by participants deliberately acting strategically prosocially to mislead their group partners and exploit the benefits of cooperation, we used an independent way to identify prosocials using the SVO measure (Block 1: 62%, Block 2: 58%). We then tested whether behaviour on the first round reflected a prosocial preference. This was confirmed: prosocials (identified through SVO) used significantly less than others: t_70_ = −3.79, *p* < 0.0001, M_prosocial_ = 11.71 (SD = 5.52), M_non−prosocial_ = 15.41 (SD = 5.05) for Block 1; t_74_ = −2.10, *p* < .05, M_prosocial_ = 12.48 (SD = 6.12), M _non−prosocial_ = 15.25 (SD = 5.00) for Block 2. We retained the classification of participants derived from observations of contributions in the first round as it was incentive compatible and not likely to be caused by strategic considerations.

Prosocials (29% in Block 1; 21% in Block 2) started with a lower usage (Block 1: M = 5.19 EUs, SD = 0.60 EUs; Block 2: M = 5.19 EUs, SD = 0.40 EUs) but we found this increased by the end of the block (Block 1: M = 15.14 EUs, SD = 5.61, *t*_20_ = −8.25, *p* < 0.001, paired-sample; Block 2: M = 11.81 EUs, SD = 7.49 EUs, *t*_15_ = −3.59, *p* = 0.003; paired-sample). Moderates (39% in Block 1, 38% in Block 2) started with an average use (Block1: M = 11.82, SD = 2.39; Block 2: M = 13.37, SD = 4.67) and slightly increased their use by the end of the block (Block 1: M = 13.54, SD = 5.11; Block 2: M = 15, SD = 5.45), which was non-significant for Block 1 (*t*_27_ = −1.68, *p* = 0.10, paired-sample) and significant for Block 2 (*t*_29_ = −2.15, *p* = 0.02, paired-sample). Selfish players (32% in Block 1; 38% in Block 2) started with a higher usage (Block 1: M = 19.43, SD = 0.89; Block 2: M = 19.79, SD = 0.61) but this was found to decrease by the end of the block: Block 1, M = 16 EUs, SD = 5.42, *t*_22_ = 3.03, *p* = 0.008, paired-sample; with a non-significant decrease in Block 2: M = 18.45 EUs, SD = 4 EUs, *t*_28_ = 1.82, *p* = 0.08, paired-sample, however, still staying above the average.

#### Retaliation and repair indices

To calculate the retaliation index across the rounds (depicted in Fig. 2b), we counted the number of participants per round who discovered that others used (on average) more than them and, of these, we calculated the proportion of participants who increased their usage on the next round (of the same block). We followed the same strategy to calculate the repair index across rounds (depicted in Fig. 2c): we counted the number of participants per round who found out that others used less than them and, of these, we calculated the proportion of participants who decreased their usage on the next round. These indices were only used for graphical representation of behaviour over the experiment.

For comparison between groups, we re-calculated each index at an individual level: e.g., how many times each participant repaired out of times they could have potentially repaired, which was then averaged across all scores in the sample. Thus the average score over the sample did not include dependent observations.

To calculate the frequency of the “turn the other cheek” events per group (Fig. 3b), we counted the number of cases, per group, when group members did not increase their usage for two consecutive rounds after finding out that others used (on average) more than them in those two rounds. For example, if a participant used 6 EUs in Round 2, and they found that on average their group used 10 EUs in Round 2, they still continued to use 6 EUs in Round 3 and Round 4. The score was averaged over the group and then the score for each group was submitted to analysis. Therefore, the analyses did not include dependent observations.

To identify individual levels of angry retaliation, we calculated the proportion of rounds for each participant when they found out that others used (on average) more than them, reported heightened anger (defined as individual mean corrected score that was higher than zero) and increased their usage on the next round, as a function of rounds when they found out that others used (on average) more than them and reported heightened anger regardless of their next move.


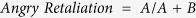


where A is a number of events when others used more electricity, which resulted in both increased anger and retaliation; B is a number of events when others used more electricity that resulted in increased anger and no retaliation.

For example, a participant found out that others used (on average) more than them on 5 rounds (out of 10 in the block) and on all those rounds they reported increased anger. However, they increased their electricity usage on the following rounds only on three occasions. In this scenario their angry retaliation score would be (3/5)*100 = 60%. A similar strategy was used to calculate the level of guilty repair:


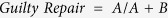


where A is a number of events when others used less electricity, which resulted in both guilt and repair; B is a number of events when others used less electricity that resulted in increased guilt and no repair.

#### Mixed-level regression modelling with social preferences

We explored whether social preferences moderate effects of unfairness on emotions, as well as emotions on energy use using a mixed-level random effects modelling approach. We used dummy variables to denote prosocial and moderate social preferences, with selfish being a reference category. Full results of regressions are reported in Supplementary Tables S2 and S3 and discussed in Supplementary Materials.

## Additional Information

**How to cite this article:** Skatova, A. *et al*. Guilty repair sustains cooperation, angry retaliation destroys it. *Sci. Rep.* **7**, 46709; doi: 10.1038/srep46709 (2017).

**Publisher's note:** Springer Nature remains neutral with regard to jurisdictional claims in published maps and institutional affiliations.

## Supplementary Material

Supplementary Materials

## Figures and Tables

**Figure 1:**
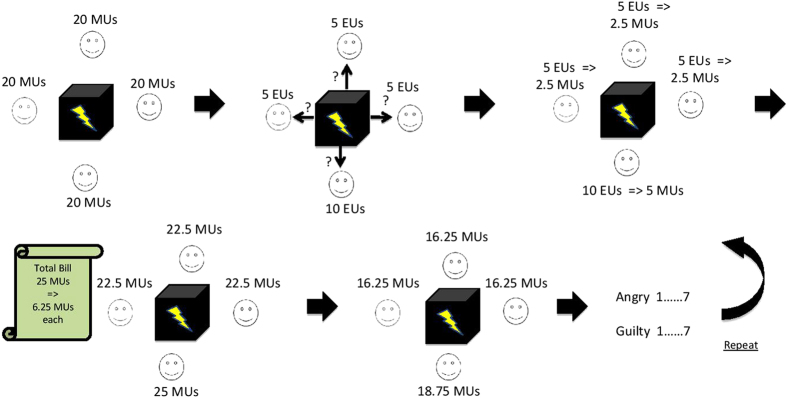
A schematic description of one round of the Shared Household Energy Use Game. Each participant in the group had an equal money endowment (20 Money Units – MUs) at the beginning of each round. Participants were required to decide (individually) how much electricity to use in the current round (ranging between 5 to 20 Electricity Units, EUs). All EUs that participants used were converted into utility based on the rate 2 EUs = 1 MU and these were added to the initial endowment. After the bill has arrived, each participant paid an equal share of the total electricity use of the group, where 1 EU cost 1 MU. Finally, participants found out (individually) about their earnings during the game and had to rate their emotional states in the current situation.
