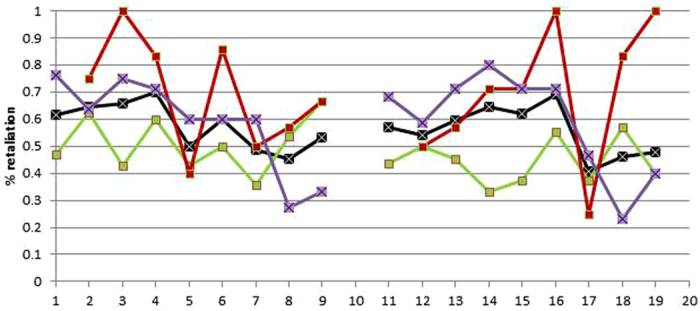# Corrigendum: Guilty repair sustains cooperation, angry retaliation destroys it

**DOI:** 10.1038/srep46899

**Published:** 2017-09-07

**Authors:** Anya Skatova, Alexa Spence, Caroline Leygue, Eamonn Ferguson

Scientific Reports 7: Article number: 46709; 10.1038/srep46709 published online: 04 27 2017; updated: 09 07 2017.

In this Article, Figure 2b is a duplication of Figure 2c. The correct Figure 2b appears below as Fig. 1.

## Figures and Tables

**Figure 1:**