# Supplementary material for: Guilty repair sustains cooperation, angry retaliation destroys it
Source: Sci Rep. 2017 Apr 27;7:46709. doi: 10.1038/srep46709 (PMC5406841; doi:10.1038/srep46709)
Supplement: Supplementary Materials [file srep46709-s1.pdf]

# Supplementary Materials

## *Guilty repair sustains cooperation, angry retaliation destroys it*

Anya Skatova*[a,b], Alexa Spence[b,c], Caroline Leygue[b,c], Eamonn Ferguson[c]

anya.skatova@gmail.com; alexa.spence@nottingham.ac.uk;
caroline.leygue@nottingham.ac.uk; eamonn.ferguson@nottingham.ac.uk

[a]Warwick Business School, University of Warwick, Coventry, UK; [b]Horizon Digital Economy Research, University Of Nottingham, Nottingham, UK; [c]School of Psychology, University Of Nottingham, Nottingham, UK

*Do emotions mediate or moderate effects of unfairness on future energy use?*

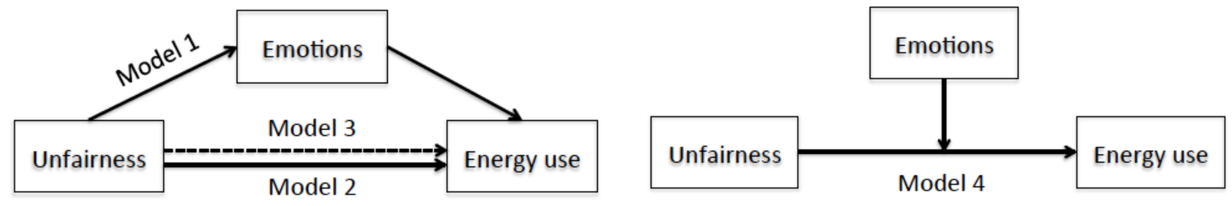


**a** **b**

**Figure S1.** Model specification to test the role of emotions on the effect of others' unfairness on future energy use. (a) Models 1, 2 and 3 test the mediating role of emotions. Dashed line represents mediated link of unfairness to energy use via emotions. (b) Model 4 tests the moderating role of emotions.

**Table S1.** Mixed-effects regression models to test mediation and moderation model in Figure S1. The table presents the results of a series of mixed-effects regression models with random intercept and slopes for round number and unfairness (N = 91) to test the mediation and moderation models in Figure S1. Emotions are person-centred. Since the analysis to predict increase in energy use was lagged, the last round in the block was excluded from all analyses including the non-lagged models (Model 1g and 1a) for comparison purposes. Number of observations is 1332 in all models. Table S1 reports unstandardized coefficients with 95% confidence intervals for fixed effects as well as random effects and model fit statistics. The model number corresponds to Figure S1 and subscripts denote (g) – guilt; and (a) – anger.

| | **Outcome variables** | | | | | | |
|---|---|---|---|---|---|---|---|
| | **Guilt$_n$** | **Anger$_n$** | **Increase in energy use on round$_{n+1}$** | | | | |
| | **Model 1$_g$** | **Model 1$_a$** | **Model 2** | **Model 3$_g$** | **Model 3$_a$** | **Model 4$_g$** | **Model 4$_a$** |
| Intercept | -0.05 (-0.18; 0.07) | 0.07 (-0.11; 0.25) | 0.32 (-0.07; 0.71) | 0.31 (-0.08; 0.69) | 0.31 (-0.07; 0.71) | 0.31 (-0.08; 0.69) | 0.15 (-0.24; 0.54) |
| Unfairness$_n$ | -0.09*** (-0.13; -0.06) | 0.14*** (0.12; 0.17) | 0.49*** (0.41; 0.56) | 0.47*** (0.39; 0.54) | 0.48*** (0.40; 0.56) | 0.47*** (0.39; 0.54) | 0.46*** (0.39; 0.54) |
| Guilt$_n$ | - | - | - | -0.24* (-0.43; -0.04) | - | -0.24* (-0.44; -0.03) | - |
| Anger$_n$ | - | - | - | _ | 0.07 (-0.10; 0.24) | _ | 0.04 (-0.13; 0.21) |
| Round number$_n$ | 0.01 (-0.02; 0.03) | -0.01 (-0.04; 0.02) | -0.04 (-0.12; 0.04) | -0.03 (-0.11; 0.04) | -0.04 (-0.12; 0.04) | -0.03 (-0.11; 0.04) | -0.02 (-0.10; 0.05) |
| Unfairness$_n$ * guilt$_n$ | - | - | - | - | - | -0.001 (-0.04; 0.03) | - |
| Unfairness$_n$ | - | - | - | - | - | - | 0.05*** |

| * anger$_n$ | | | | | | | (0.03; 0.08) |
|---|---|---|---|---|---|---|---|
| **Random effects** | | | | | | | |
| Intercept σ (participant) | 0.16 | 0.39 | 0.21 | 0.14 | 0.18 | 0.14 | 0.14 |
| Round (slope) | 0.01 | 0.01 | 0.01 | 0.01 | 0.01 | 0.01 | 0.01 |
| Unfairness (slope) | 0.02 | 0.01 | 0.05 | 0.05 | 0.05 | 0.05 | 0.05 |
| **Model fit** | | | | | | | |
| Deviance (-2LL) | 3742.7 | 4249.9 | 7261 | 7255.6 | 7260.4 | 7255.6 | 7246.3 |

**** p < .001, p < .01, * p < .05, ' p < .01*

In the mixed-level random intercept model, the negative value of unfairness score (i.e., when others use less than the participant) significantly predicted guilt (B = -0.09, 95% CI: [-0.13; -0.06], $p < .001$, Model 1g, Table S1), while unfairness of others (positive value of unfairness score - others use more than the participant) significantly predicted anger (B = 0.14, 95% CI: [0.12; 0.17], $p < .001$, Model 1a, Table S1) over and above effects of the round number, which was non-significant for both guilt and anger. These analyses suggest that when participants saw that others were unfair, they reported that they were angry, while when others used less, participants reported they felt guilty.

Next, unfairness of others significantly predicted an increase in energy use on the round$_{n+1}$: B = 0.49, 95% CI: [0.41; 0.51], $p < .001$, with no direct effect of the round number, Model 2, Table S1. Experienced unfairness remained a significant predictor of subsequent energy use both when accounting for anger (B = 0.48, 95% CI [0.40; 0.56], $p < .001$, Model 3a, Table S1) and guilt (B = 0.47, 95% CI [0.39; 0.54], $p < .001$, Model 3g, Table S1). Further, guilt significantly predicted a decrease in energy use on the next round: B = -0.24, 95% CI [-0.43; -0.04], $p = .018$, while anger had no direct effect on increase in energy use on the next round: B = 0.07, 95% CI: [-0.10; 0.24], ns.

We further tested whether there is a moderation of unfairness by emotions: effects of unfairness on increase in energy use were moderated by anger (B = 0.05, 95% CI: [0.03; 0.08], $p < .001$, Model 4a, Table S1), with a significant improvement in model fit when accounting for anger by unfairness interaction (deviance index changed from 7260.4 to 7246.3, $p < .001$). Thus, the effect of unfairness was stronger when the individual simultaneously felt more anger. The effect of unfairness on decrease in energy use was not moderated by guilt, as entering the guilt by unfairness interaction did not significantly improve the fit of the model (deviance index did not change from 7255.6).

Since anger did not have a direct effect on increase in use, while guilt had a direct effect on decrease in use, we only tested mediation effects of guilt. The analysis showed that guilt mediated effects of discrepancy between participants and others use of energy use with proportion mediated 0.04, 95% CI: [0.01; 0.09], $p < .001$.

*Does the effect of guilt on energy use differ between prosocials, moderates and selfish players?*

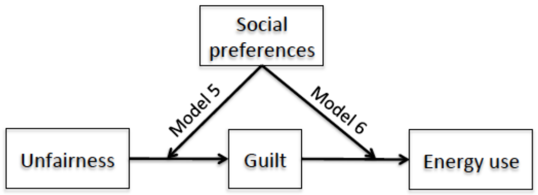


**Figure S2.** Regression models with social preferences (prosocial, moderates or selfish) moderating the effects unfairness on guilt and guilt on energy use.

**Table S2.** Mixed-effects regression models to test the model in Figure S2. The table presents the results of a series of mixed-effects regression models with random intercept and slopes of round number and unfairness (N = 91) predicting increase in usage on the round$_{n+1}$ and guilt after round$_n$ to test moderation models shown Figure S2. Emotions are person-centred. Since the analysis to predict increase in energy use was lagged, the last round in the block was excluded from all analyses including the non-lagged model (Model 5) for comparison purposes. Number of observations is 1332 in all models. Table S2 reports unstandardized coefficients with 95% confidence intervals for fixed effects as well as random effects and model fit statistic. Social preferences are defined as selfish (those who used 18, 19 or 20 EUs on Round 1), which served as a reference category for moderates (those who used between 8 and 17 EUs on Round 1) and prosocials (those who used 5, 6 or 7 EUs on Round 1).

| | **Outcome variables** | |
|---|---|---|
| | **Guilt$_n$** | **Increase in energy use on round$_{n+1}$** |
| | **Model 5** | **Model 6** |
| Intercept | -0.16 (-0.35, 0.02) | 0.52' (-0.01; 1.05) |
| Unfairness$_n$ | -0.06** (-0.11; -0.02) | 0.46*** (0.35; 0.57) |
| Guilt$_n$ | - | 0.35' (-0.02; 0.71) |
| Moderates | 0.11 (-0.09; 0.31) | -0.31 (-0.92; 0.28) |
| Prosocials | 0.25* (0.02; 0.49) | -0.56 (-1.30; 0.14) |
| Round number$_n$ | 0.01 (-0.01; 0.03) | -0.02 (-0.10; 0.06) |
| Guilt$_n$ * Moderates | - | -0.87*** (-1.33; -0.41) |
| Guilt$_n$ * Prosocials | - | -0.67* (-1.23; -0.10) |
| Unfairness * Moderates | -0.09** (-0.16; -0.03) | -0.04 (-0.20; 0.12) |
| Unfairness * Prosocials | 0.01 (-0.07; 0.08) | 0.09 (-0.08; 0.27) |
| | **Random effects** | |
| Intercept σ (participant) | 0.18 | 0.11 |
| Round (slope) | 0.003 | 0.01 |
| Unfairness (slope) | 0.01 | 0.04 |
| | **Model fit** | |
| Deviance (-2LL) | 3726.5 | 7238.4 |

**** p < .001, p < .01, * p < .05, ' p < .10*

We tested whether the effects of guilt on increase in energy use were different for participants with different social values – prosocial, moderates and selfish. Results demonstrated that prosocials

reported more guilt (B = 0.25, 95% CI: [0.02; 0.49], $p$ = .03) in general, while moderates reported more guilt after being unfair to others: B = -0.09, 95% CI: [-0.16; -0.03], $p$ < .001 (see Table S2, Model 5). The effect of guilt on decrease in energy use was significant for prosocials (B = -0.67, 95% CI: [-1.33; -0.41], $p$ = .02) and moderates (B = -0.87, 95% CI: [-1.23; -0.10], $p$ < .001) when profit-seekers were treated as a reference group. This suggests that the effect of guilt on energy use was moderated by differences in social preferences, with the effects of guilt on energy use moderated by prosocial and moderate social preferences.

*Does moderation effect of anger differ between prosocials, moderates and selfish players?*

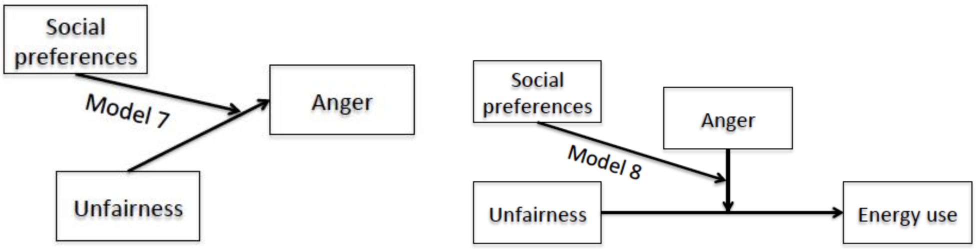


**a** **b**

**Figure S3.** (a) Moderation model of the effects of unfairness on anger conditioned on social preferences: prosocial, moderates or profit-seekers; (b) Moderation model of the effects of unfairness on energy use, conditioned on unfairness by anger and social preferences.

**Table S3.** Mixed-effects regression testing the models in Figure S3. The table presents the results of a series of mixed-effects regression models with random intercept and slopes of round number and unfairness (N = 91) predicting increase in usage on the $round_{n+1}$ and anger after $round_n$ to test moderation models shown Figure S3. Emotions are person-centred. Since the analysis to predict increase in energy use was lagged, the last round in the block was excluded from all analyses including the non-lagged model (Model 7) for comparison purposes. The number of observations is 1332 in all models. Table S3 reports unstandardized coefficients with 95% confidence intervals for fixed effects as well as random effects and model fit statistics. Social preferences are defined as selfish (those who used 18, 19 or 20 EUs on Round 1), which served as a reference category for moderates (those who used between 8 and 17 on Round 1) and prosocials (those who used 5, 6 or 7 on Round 1).

| | **Outcome variables** | |
|---|---|---|
| | **$Anger_n$** | **Increase in energy use on $round_{n+1}$** |
| | **Model 7** | **Model 8** |
| Intercept | 0.39*** (0.18; 0.60) | 0.29 (-0.26; 0.85) |
| $Unfairness_n$ | 0.12*** (0.08; 0.16) | 0.43*** (0.32; 0.55) |
| $Anger_n$ | - | -0.01 (-0.29; 0.26) |
| Moderates | -0.44** (-0.67; -0.22) | -0.10 (-0.75; 0.52) |
| Prosocials | -0.57*** (-0.83; -0.32) | -0.16 (-0.95; 0.58) |
| Round $number_n$ | -0.01 (-0.04; 0.02) | -0.03 (-0.10; 0.05) |
| $Anger_n$ * Moderates | - | 0.36’ (-0.04; 0.76) |
| $Anger_n$ * Prosocials | - | 0.11 (-0.35; 0.56) |
| Unfairness * $Anger_n$ | - | 0.12*** (0.08; 0.16) |
| Unfairness * Moderates | 0.06’ (-0.001; 0.12) | -0.01 (-0.17; 0.15) |
| Unfairness * Prosocials | 0.03 (-0.03; 0.10) | 0.14 (-0.04; 0.31) |
| Unfairness * $Anger_n$ * Moderates | - | -0.09* (-0.17; -0.02) |
| Unfairness * $Anger_n$ * Prosocials | - | -0.17*** (-0.24; -0.10) |

| | Random effects | |
|---|---|---|
| Intercept σ (participant) | 0.28 | 0.20 |
| Round (slope) | 0.01 | 0.04 |
| Unfairness (slope) | 0.01 | 0.004 |
| | Model fit | |
| Deviance (-2LL) | 4224.1 | 7212.4 |

**** p < .001, p < .01, * p < .05, ʹ p < .10*

We tested whether the effects of unfairness on anger and the effects of unfairness by anger on energy use were different for participants with different levels of social preferences. Moderates and prosocials reported less anger than selfish: B = -0.44, 95% CI: [-0.67; -0.22], *p* < .001 and B = -0.57, 95% CI: [-0.83; -0.32], *p* < .001, respectively. Unfairness of others had a marginal effect on anger for moderates: B = 0.06, 95% CI: [-0.001; 0.12], *p* = .06. However, moderates and prosocials used less energy when others were unfair and when feeling angry: they increased the energy use less: B = -0.09, 95% CI: [-0.17; -0.02], *p* = .04 and B = -0.17, 95% CI: [-0.24; -0.10], *p* < .001, with an overall moderation of unfairness by anger remaining significant: B = 0.12, 95% CI: [0.08; 0.16], *p* < .001.
